# Increasing Internal and External Validity Through a Multimethod Evaluation With Qualitative Comparative Analysis (QCA) and Process Tracing: The Case of Training Transfer Effectiveness in Flemish SMEs

**DOI:** 10.1177/0193841X251352020

**Published:** 2025-06-25

**Authors:** Priscilla Alamos-Concha, Valèrie Pattyn, Bart Cambré, Benoît Rihoux

**Affiliations:** 1 Radboud University, Nijmegen, Netherlands; 2 4496 Leiden University, Leiden, Netherlands; 3 452561 University of Antwerp and Antwerp Management School, Antwerp, Belgium; 4 University of Louvain, Louvain-la-Neuve, Belgium

**Keywords:** qualitative comparative analysis, process-tracing, multimethod evaluation

## Abstract

This paper presents a methodological advancement by integrating Qualitative Comparative Analysis (QCA) and Process Tracing (PT) in the evaluation of training transfer effectiveness in Flemish SMEs. This multimethod approach leverages the strengths of both QCA and PT to enhance internal and external validity, offering a robust framework for capturing the conditions and causal mechanisms underlying policy interventions. By sequentially applying QCA to identify necessary and sufficient conditions and PT to unpack the causal processes, the study provides a comprehensive analysis that addresses both “what works” and “how it works.” Our findings demonstrate that combining these methods allows for more nuanced insights into the effectiveness of training programs, ultimately contributing to the empirical validation of policy theories and the development of evidence-based interventions. This research underscores the potential of multimethod evaluations to produce more reliable and generalizable results, thereby offering valuable guidance for evaluators and policymakers seeking to enhance the impact of their programs.

## Introduction

Recent methodological overviews or toolboxes in evaluation communities (e.g., HM Treasury, 2020; Vaessen et al., 2020) now commonly include references to Qualitative Comparative Analysis (QCA) and Process Tracing (PT) as valuable methods to capture in which conditions and how interventions work. Accordingly, the number of evaluations in which QCA (e.g., Blackman et al., 2013; Krupnik et al., 2023; Pattyn et al., 2019; Verweij & Gerrits, 2013) or PT (e.g., Befani & Stedman-Bryce, 2017; Raimondo, 2020; Wauters & Beach, 2018) are used is also steadily growing (see also Lemire et al., 2020). Multimethod studies in which both methods are combined in a single evaluation remain rare, however, making the potential of such a combination relatively unexplored. In the design that we are developing here, QCA and PT are jointly used at the data analysis stage, and both methods share some common “case based” epistemological foundations. We therefore label such a design as being “multimethod,” which is a more precise label—a subset—within the broad range of “mixed methods” designs (Rihoux et al., 2021, p. 186; Johnson, Onwuegbuzie, & Turner, 2007).

An evaluation sequentially resorting to QCA and PT provides the strong advantage to combine cross-case analysis with in-depth within case analysis and can as such increase confidence in the causal process linking condition(s) and a particular outcome (Pattyn et al., 2022; Rihoux et al., 2021). Besides, a rigorous combination of both methods can also increase the external validity of evaluation findings, which is especially relevant when one wishes to empirically validate the scope of cases to which process-level generalizations can be made (Beach et al., 2022). However, such a multimethod protocol does not come without any challenges, and a well thought out strategy is needed.

In this article, we provide concrete guidance on how one can combine QCA and PT in a single evaluation. Importantly, we apply a *system-oriented approach* to PT which is inherent to PT as it aims to unpack detailed causal mechanisms. In this approach, causal processes (aka causal mechanisms) are understood as systems of interacting parts within which the activities of actors transfer causal forces from causes (e.g., a policy intervention) to outcomes (e.g., a particular societal effect) (Beach & Pedersen, 2016; Machamer, 2004; Machamer et al., 2000; Waldner, 2014). Such an approach differs from a more minimalist and counterfactual conceptualization of process-tracing, which does not unpack how the different parts of a mechanism actually act together in linking the cause with the outcome (Beach & Pedersen, 2019, p. 246). For policymakers, or commissioners of an evaluation study, investing in an in-depth understanding of the mechanism accountable for a particular outcome is particularly useful, not least to expand the evidence base of robust policy theories. While there are a few specialized works on multimethod research (MMR) in which QCA and PT are combined (e.g., Schneider & Rohlfing, 2019), these do not apply a system-oriented approach to PT. This article addresses this gap and shares important lessons for evaluators and commissioners of evaluations who wish to benefit from a more holistic QCA-PT multimethod design.

To do so, we draw insights from an evaluation study commissioned by the European Social Fund (ESF) Agency in Flanders (Belgium) and implemented by the authors of this paper (Alamos-Concha et al., 2022). The purpose of the evaluation was to explain and understand under which conditions and how ESF-subsidized training programs produce impact. Impact, in our evaluation, is conceived as the transfer of the learned skills to the workplace. This dual evaluation purpose is reflective of the multimethod approach applied: with QCA we investigated the core combinations of conditions under which the program was successful, whereas PT enabled us to unravel how the program actually worked. This combination, as we demonstrate via this evaluation, has the potential to contribute to mid-range theorizing with relevance for other organizations and training programs.

This article is structured as follows. First, we concisely discuss the respective value of QCA and PT for evaluation research and elaborate on the potential of combining both in a single study. Next, we explain how one can approach the multimethod combination in practice, illustrating via the above mentioned evaluation of training transfer effectiveness. We conclude the article with a reflection on what the combination of QCA and PT has to bring for internal and external validity.

## The Value of QCA and Process Tracing for Evaluation Research

Given the complexity of policy making and social realities, a policy intervention often results in different outcomes (effects or impact) depending on the context in which it is implemented. This is particularly also one of the assumptions underpinning realist evaluation (Befani & Sager, 2006). QCA aligns with this approach and enables one to identify the (combination of) condition(s) that are *necessary* and/or *sufficient* for a particular outcome to occur. A (combination of) condition(s) that qualifies as necessary will always be present/absent whenever the outcome is present/absent. Conversely, it is considered sufficient if the outcome appears whenever the condition is present.

From this set-theoretic logic, it follows that a policy intervention may be only a part of a causal package that is sufficient to produce a certain effect (conjunctural causation), and there may be different such packages that can trigger the same effect (equifinality) (Stern et al., 2012). At the same time, if a certain condition is relevant for the outcome in a particular setting, the absence of this condition does not imply that the outcome will also be absent (asymmetric causality). QCA is designed to deal with these elements of configurational complexity (Berg-Schlosser et al., 2009). Given its emphasis on the conditions under which an intervention worked, the method is well-suited for *ex post* or *in itinere* evaluations with a learning rather than an accountability objective. It also lends itself both to policy theory testing and to more exploratory policy theory development (Pattyn, 2023).

In contrast to experimental designs that focus on “what works” questions, it should be clear that in QCA evaluations, the evaluator’s interest is not in the average effect or difference that an intervention makes, but rather in the varied performance of an intervention in different settings (Befani, 2016), regardless of whether a setting is an outlier (Pattyn et al., 2022). These outlier settings may in fact be as relevant for policy makers or other evaluation stakeholders as more mainstream implementation contexts.

QCA includes a range of techniques that allow for the systematic comparison of cases (e.g., beneficiaries of a policy intervention, such as individuals or organizations) across settings in a transparent and replicable way. A prerequisite for its application, consistently with its set-theoretic underpinnings, is that the conditions and the outcome need to be calibrated to enable a systematic comparison across cases. In crisp set QCA (csQCA), the original version, a researcher will use binary scores with 1 referring to the presence (or high, or similar), and 0 referring to its absence (or low or similar). Crucially, 1 and 0 express qualitative differences in kind. In the fuzzy set variant of QCA (fsQCA), cases can have partial membership in a set and have any score between 0 and 1, considering that empirical manifestations of social phenomena can differ in degree (Schneider & Wagemann, 2012, p. 14). More details on the operational steps of a QCA research cycle can be found in QCA specific evaluation manuals (e.g., Befani, 2016).

Despite its potential to identify patterns of necessity and sufficiency in the causal complexity characterizing many policy interventions, QCA will not help us understand “*how*” a particular effect was produced. Addressing such a question is what PT is designed for as a method. To put it in simple terms: PT enables evaluators to open the black box between a particular (combination of) condition(s) and a particular outcome, and it unpacks the mechanism that links the elements together in a particular context. The system-oriented version of PT resonates especially well with theory-based evaluation approaches, and with realistic evaluation in particular (Pawson, 2008; Stame, 2004; Stern et al., 2012). The core idea is that a causal relationship will only occur when triggered by a so-called generative mechanism (Pawson, 2008). How this mechanism manifests itself and to which outcomes it will lead to will depend on the broader context, as expressed by the CMO (Context-Mechanism-Outcome) configuration with which realistic evaluation is often associated (Pawson, 2008).

To identify and test the operation of a mechanism in a particular context, evaluators need to start from a process theory of change (ToC) (Raimondo, 2023), which details the hypothesized relationship between the configuration of conditions and the outcome in a fine-grained way. It requires being very specific about how entities (such as actors or organizations) are expected to engage in activities in all parts of the mechanism. The activities are to be conceived as the producers of change, transmitting causal forces (Beach & Pedersen, 2019, p. 38). After this hypothesis-building, the evaluator must articulate which “fingerprints” (Beach & Pedersen, 2019) could be left in all parts of the mechanism, which can serve as a confirmatory signature (Raimondo, 2023) that a given action and linkage took place. Finally, the corresponding evidence will need to be traced (for more extensive details, see, e.g., Beach & Pedersen, 2019).

By rigorously reconstructing and testing a hypothesized causal mechanism linking a (combination of) condition(s) and an outcome in a real case, PT is strong in terms of internal validity. Nonetheless, such an in-depth within-case analysis comes at the cost of making conclusions beyond the analyzed cases. Combining QCA with PT offers the advantage of leveraging the external validity of the mechanistic conclusions to cases sharing similar combinations of conditions. With QCA, one can gain cross-case knowledge about the population of cases, which can help understand the combination of conditions, and thus the contextual boundaries, in which a given mechanism can be operative (Beach & Pedersen, 2019, p. 6). Combining PT with QCA thus makes PT not only more holistic, but also more robust (Alamos-Concha et al., 2022). Additionally, via this QCA-PT multimethod design, one may contribute to middle-range theorizing about the operation of a mechanism in specific settings. In what follows, we detail how we developed and implemented the QCA-PT design in practice.

## Combining QCA and Process Tracing in Practice

### The Case of Evaluating Training Transfer Effectiveness in Flemish SMEs, and the Choice for a QCA-PT Design

To explain how to go about a QCA-PT evaluation in practice, we rely on an evaluation of training transfer effectiveness of Flemish (Belgian) Small and Medium-sized Enterprises (SMEs) of training programs subsidized by the Flemish ESF Agency (2017–2020). This Agency also commissioned the evaluation. The overarching research aim was to unravel the factors and processes driving training transfer effectiveness (outcome), which we defined as the “*application or use of knowledge (content, skills or attitudes) acquired in a training program to the job by trainees*” (TRANSFER). Based on specialized training transfer and educational literature, eight conditions were specified that could be expected to contribute to further training transfer: Peer support (PEERSUP), Supervisor support (SUPERV), Relapse prevention-goal setting (RELAPSE), Sense of urgency (SURG), Identical elements with training (IDENT), Training program as active learning method (TRAPO), Autonomy (AUTO), and absence of workload (NONWL).

Our evaluation focused on two specific questions:

(1) Under which combination of conditions do employees succeed to transfer learned social skills to the workplace?
(2) How and when do employees succeed to transfer learned social skills to the workplace?

Whereas QCA is suited to answering the first research question, PT is capable of addressing the second one. The data collection consisted of two parts. In view of the QCA analysis, a survey was sent to the trainees before and after the training, to measure differences in learning and retention. 50 respondents as cases of training transfer effectiveness in nine Flemish firms from 2018 to 2020 were eventually kept in the final analysis. Respondents who did not participate in both survey rounds or from whom we only had partial responses were excluded. For the PT part, we conducted semi-structured interviews with nine key stakeholders responsible for the respective subsidized training programs, and held a range of in-depth interviews with eight trainees for whom we observed training success, that is, effective transfer.

Whereas one could as well have opted for the inverse approach, we chose a “*QCA-first*, *then PT*” sequence. Beginning with QCA helped us reduce the complexity of the surveyed literature, by identifying the core conditions that are necessary and/or sufficient for training transfer effectiveness. Additionally, it enabled us to make an informed case selection for the in-depth analysis with PT, thereby modestly enhancing the external validity of the findings to similar contexts.

### QCA in a Multimethod Evaluation with Process Tracing (QCA-PT)

**Figure 1.**
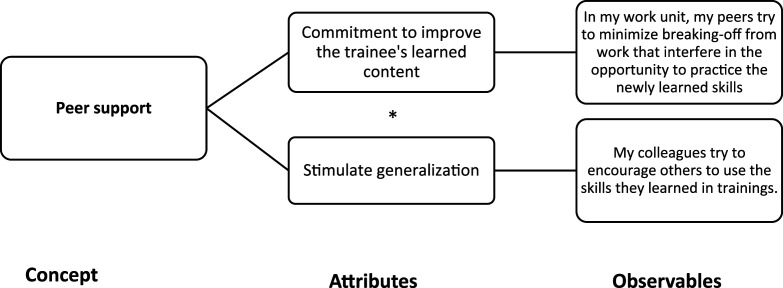
Conceptualization and operationalization of “peer support” concept.

As previously mentioned, our design followed the “*QCA-first*, *then PT* sequence,” but we adopted a mechanistic approach to causality throughout the whole evaluation. This strategy is part of the methodological alignment that we advocate following Beach and Pedersen (2016) and our own experience with the evaluation process (Pattyn et al., 2022). Several measures were taken during the QCA stage of the research in anticipation of the subsequent in-depth case studies (see Figure 1). With these measures, we primarily aimed to reduce the risk of mechanistic heterogeneity, acknowledging the possibility that the same cause can be linked to the same outcome through different independent processes. From a causal inference point of view, mechanistic heterogeneity can be most problematic for external validity.

**Figure 2.**
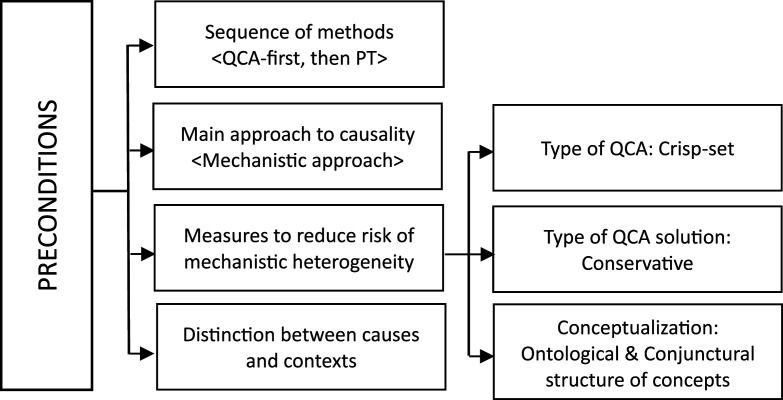
Preconditions for within-case selection.

Four specific measures were therefore taken (see Figure 2). First, we deliberately applied the crisp set QCA (csQCA) variant to our evaluation. The risk of mechanistic heterogeneity that impedes the generalization of findings can be intrinsically higher when using fuzzy set QCA (fsQCA). In fsQCA, one has to deal with differences in degree that are perhaps not so much relevant at the cross-case level, but that can produce causal heterogeneity at the level of mechanisms. One can as such not rule out the possibility that a causal mechanism operates differently for a particular group of cases covered by the same solution. It may also be that different mechanisms exist depending on different case scores within conjunctions and outcome (Beach & Pedersen, 2019). To reduce such a risk to a minimum (though it cannot completely rule out), we proceeded with csQCA, where the presence and absence of conditions is clearly delimited.

Second, we carefully conceptualized our conditions and outcome in an essentialist way (Goertz, 2006). Thus, we focused on ontological attributes that possess causal powers to trigger causal mechanisms (conditions) or that have the capacity to receive some impact from the causes and mechanisms (outcome) (Beach & Pedersen, 2016, 2019). We then opted for a classical conceptualization of concepts following a “conjunctural” logic (i.e., forming concepts by exploiting the logical AND). We deliberately avoided multi-attribute concepts (i.e., which are formed with logical OR and which act as substitutes), as such attributes that seem similar at cross-case level can potentially trigger different mechanisms at within-case level (see Pattyn et al., 2022 for more details).

As an illustration, Figure 1 shows the application of this configurational approach to the conceptualization of the “peer support” concept. The concept contains two theoretical attributes that jointly constitute it (“*” stands for the logical AND).

Third, we distinguished between contexts and causal conditions (Beach & Pedersen, 2016, 2019; Pattyn et al., 2022; Alamos-Concha et al., 2021). In our definition, contexts are passive and do not trigger processes; rather they determine whether a causal relationship functions as theorized. Causal conditions, in contrast, are active and trigger process-level causal explanations. These explanations provide an account of what actors are doing, explaining why the actors’ activities are linked together and how they contribute to producing the outcome in a particular case (see more in section 4).

Finally, we opted for the QCA “conservative” solution (i.e., the longer, less parsimonious solution) to avoid the risk of excluding conditions that jeopardize the well-functioning of the causal process. A mechanism may well be operative within a certain context but may disappear or operate differently when selecting the “parsimonious” or “intermediate solution.” This could then imply that the causal process is not working as expected (Beach & Pedersen, 2016, 2019). In essence, the exclusion of conditions might change the causal dynamics between the conditions and the outcome and is thus preferably avoided by safely opting for the typically longer and more conservative QCA solution (Alamos-Concha et al., 2022, provide the extensive explanation); see Figure 2.

With these precautionary measures in mind, we present here below the conservative solution for our case example. It includes eight sufficient terms, each term consisting of a conjunction of certain conditions. All eight terms exhibit high consistency and PRI (Proportional Reduction in Inconsistency), which serves as an alternative metric in social research for evaluating the consistency of subset relations. Additionally, they have relatively low unique coverage values, while the overall solution coverage remains comparatively high (Schneider & Wagemann, 2012). Following Table 1, we can observe that several (partly) distinct pathways lead to training transfer effectiveness (TRANSFER). There was not a single case in which all conditions were favorable. There were a couple of similarities though. For instance, most of those individuals who successfully transferred their training programs were taught with an active learning method (TRAPO). However, it’s surprising to note the lack of sense of urgency of the trainees (SURG)—as it only appears in three pathways. Additionally, peer support (PEERSUP) was relevant for the successful transfer in 4 out of 8 pathways. Relapse prevention and goal setting (RELAPSE) seem to play a modest role for the successful outcome. The identical elements (IDENT) and balanced workload (NONWL) contexts seem to have played a quite relevant role—rather as facilitators. Finally, the role of the supervisor (SUPERV) seems to play a modest role in most of the pathways.

**Table 1.** Conservative solution—QCA.

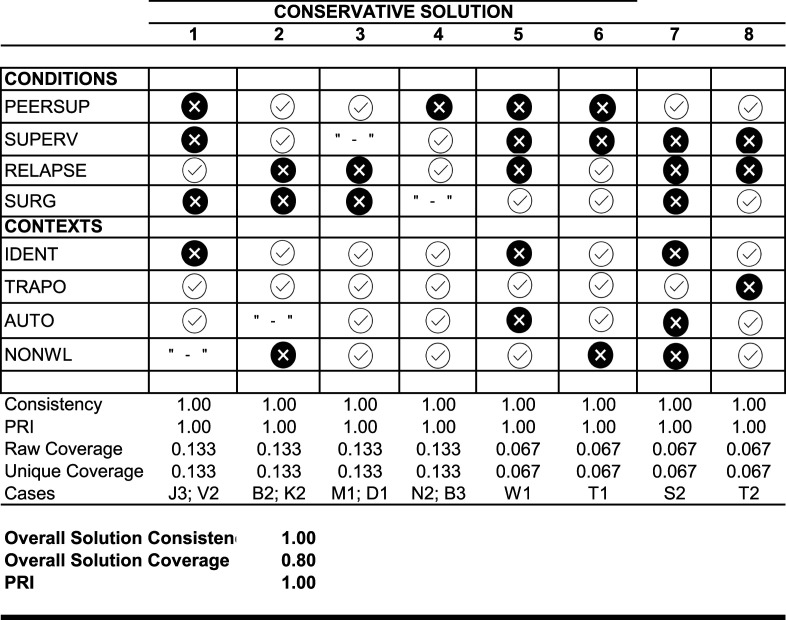

Note: The white checked circles indicate “presence of the condition,” and black crossed circles indicate the “absence of the condition.” Anything else is not relevant for the analysis.

Zooming in on a particular pathway (path 4), we examine the cases covered by this configuration, in particular cases N2 and B3. In these cases, we can observe that peer support was not needed as long as the supervisor provided strong support or when the trainee had a sense of urgency to learn and transfer or when the trainee implemented techniques of relapse prevention-setting goals. In N2 and B3, the training transfer effectiveness was thus characterized by a lack of peer support within the contexts of autonomy, balanced workload, training program as an active learning methods and identical elements. It may be worth noting that sense of urgency was irrelevant since its presence or absence does not make any difference in the impact on transfer in those cases.

In an effort to back to the cases, coincidently, both cases work at the same company. They both experienced support by their supervisor and there was relapse prevention and employee goal setting. Although, based on the survey, it was concluded that they did not experience clear peer support, during the interviews with these two employees, they did mention that they received some peer support. This can be explained by the concept structure we follow in this multimethod design. It is not enough to mention the support by peers if all the attributes of the concepts are not being considered. Additionally, we observed that in N2, the effect of supervisor support on training transfer was quite clear, while with B3 we could see how employee goal setting and relapse prevention contribute better to training transfer.

Beyond the theoretical contribution of our QCA findings, we can also elaborate on those findings that may enable evaluators to identify those core combinations of conditions that together contribute to a training transfer effectiveness. We can say that the peer and supervisor support combined with the absence of a sense of urgency and relapse prevention-goal setting, within the contexts of identical elements, training program as active learning methods, and a balanced workload, influence training transfer effectiveness (path 2).

Conclusions can also be formulated at the individual or small group level, with a focus on those conditions that enable groups to take action. For instance, our findings reveal that trainees are capable of transferring training skills in a supportive environment, even if they do not feel a sense of urgency or do not engage in relapse prevention. At the organizational level, we can explore which conditions enable groups to take action, with a balanced workload being the key factor. Finally, at the level of the training program, we can elaborate on the identical elements of the training and training programs as active learning methods to pave the way to better transfer the learned content to the job.

While these findings reveal what worked in the cases explained by the different pathways, thereby generating external validity (and middle-range theorizing), the actual causal process producing training transfer effectiveness can only be unpacked by opening the black box linking the conditions with the outcome. This is where PT comes in.

### Process Tracing in a Multimethod Research with QCA (QCA-PT)

QCA informs us about the (combinations of) conditions under which the training transfer effectiveness occurs, but it does not explain how the transfer takes place. How can we validate and increase our confidence in the existence of a causal process linking these conditions with the outcome? Would it be even possible to generalize the process to other cases beyond the ones we observed? These are the questions at stake in this section. We show several appropriate scenarios to consider, without claiming to be exhaustive.

**Figure 3.**
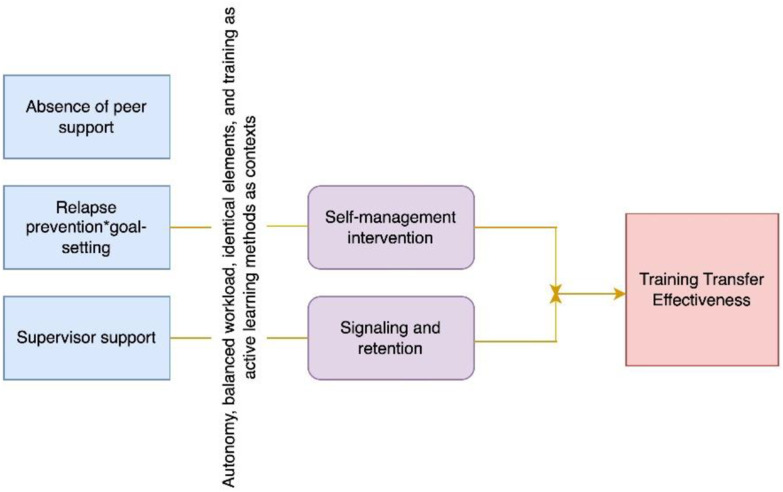
Potential causal process linking causes and outcome.

Validating the presence of a causal process linking conditions with an outcome is at the core of within-case analysis. This basically requires getting strong evidence of the processual linkages that enable robust causal mechanistic inferences. Of course, there can be several mechanisms potentially linking the combination of conditions with training transfer effectiveness as an outcome. Figure 3 presents two such potential mechanisms: “self-management intervention,” and “signaling and retention” in a two-way conjunction. The former rather relates to bottom-up behavior, in which employees are themselves active in the learning and performance stages of training. The latter is more top-down oriented, with the supervisor or superior facilitating the process of retention and motivating the transfer process. While these are two different mechanistic claims analytically speaking, they can both be compatible when acting in parallel, together, or in sequence. It is only via a systematic study of the mechanisms that one can establish how the process exactly operates, and how the mechanisms potentially interact.

To do so, consistent with a system-level understanding of causal mechanisms, evidence needs to be collected for each part of the process, and for the actual interactions between social actors or entities engaging in activities (Machamer et al., 2000). This is the only way to validate or increase confidence about the empirical existence of a theorized mechanism in a particular case. For the “self-management intervention” mechanism and its constituent parts, a system-level approach to process tracing is presented in Table 2.

**Table 2.** Example of an In-Depth PT Structure.

Configuration		Signaling and Retention Causal Mechanism (system)							OUTCOME
Peer support* RELAPSE PREVENTION-GOAL SETTING* SUPERVISOR SUPPORT	Theory	Part 1 Actor-activity	Part 2 Actor-activity	Part 3a Actor-activity Part 3 b Actor-activity	Part 4 Actor-activity	Part 5 Actor-activity	Part 6 Actor-activity	Part 7 Actor-activity	Training transfer effectiveness
	Empirics	Part 1 Actor-activity	Part 2 Actor-activity	Part 3a Actor-activity Part 3 b Actor-activity	Part 4 Actor-activity	Part 5 Actor-activity	Part 6 Actor-activity	Part 7 Actor-activity	

To know how this causal process unfolds in a particular setting, evidence needs to be gathered for each of the activities carried out by entities that transmit causal forces to the next actor in producing training transfer effectiveness.

The question is then: which cases deserve such in-depth investigation and can provide this evidence, also knowing that it is often only realistic to engage in a detailed tracing of a mechanism in a very limited number of cases? We should stress that it is important to proceed from the QCA outputs, and in particular with the pathways (terms) that are most in line with one’s analytical interests. To understand how a process takes place, “typical cases” are more relevant, because they are members of the configurations and the outcome. To this end, we applied the test corridor technique (Schneider & Rohlfing, 2019) for crisp-set QCA: “*With crisp sets, we know that a typical case’s membership in FC*^1^ *[focal conjunct] and Y is 1. We expect this case’s membership in the mechanism M to be 1 as well because only then are both parts of the sequence FC ⇒ M ⇒ Y empirically consistent*” (Schneider & Rohlfing, 2019, p. 264).

In principle, different types of cases lend themselves to validate the existence of a given process, depending on the purposes of one’s research. Assuming that theory-building or theory-testing is at stake, it is advisable to select a single typical case. In such a case, one would expect to see the hypothesized causal mechanism operating, i.e. empirically linking the configuration (term) and the outcome.

Applying this to our case example, and considering the conservative solution (Table 1), case B3 could serve as a typical case for training transfer effectiveness. Term 4 of the solution explains this case and indicates that the “absence of peer support” combined with “supervisor support” and “relapse prevention-goal,” and “identical elements,” and “training programme as an active learning method” and “autonomy,” lead to training transfer effectiveness. Note that the last four conditions act as contexts which facilitate the production of transfer and which affect the functioning of the mechanism. In contrast to causal conditions which are capable to trigger a mechanism, contexts are rather passive in their productivity, but need to be present for the correct functioning of the mechanism (Pattyn et al., 2022, p. 40).

On the basis of substantive literature on training transfer effectiveness, we have identified the “self-management intervention” causal process which can link these particular conditions with the outcome. Table 3 details the constituent parts of the mechanism. The actual evidence collected to verify these different parts consisted in a series of interviews with the case respondents, and in the analysis of fine-grained secondary data. Our findings confirmed that goal setting was indeed present in B3. We equally observed how the implementation of the training turned out to be beneficial for B3 in doing a better job. More specifically, and related to the type of training, the training helped B3 expressing attention for self-set goals (see Part 1—hereafter P1). This subsequently supported B3 to fully orient themselves toward achieving specific learning and performance goals (see P2). In turn, this boosted B3’s motivation to apply the training and proved useful to develop several ways to achieve these goals. B3 also clearly kept these goals in mind and asked questions where needed (see P3). A clear orientation toward the goals made them avoid potential threats in applying the training (see P4). This also made them consider alternative scenarios to use the training. Being aware of potential obstacles, B3 engaged in discussions and actively considered certain techniques or strategies to overcome them (see P5a). In parallel, they set up a support network to assist in transferring the training to the job (see P5b). The trainee further anticipated potential slips based on previous experiences (see P6), which helped them apply a suitable coping strategy. The training effectively helped B3, after a conflict had arisen, to talk about the issues at hand with peers (see P7). All of this led them monitor their own performance and create self-rewards (P8). Paying this much attention to the implementation of the training resulted in the successful application of the training for B3.

**Table 3.** Causal Mechanism “Self-Management Intervention.”

	Cause: Goal setting AND Relapse prevention	Provide direction for attention		Mobilizing effort	Pros and cons generalization
		Part 1	Part 2	Part 3	Part 4
Theorization	Formulation of training goals by employees AND commitment to overcome the obstacles when addressing difficulties in applying new knowledge at work.	Based on the new training skills to be acquired, the trainees identify some kind of goals [distal and proximal] (either specific, challenging or difficult) to help them with expressing attention.	With this knowledge in mind, the trainees organize their effort either focused on learning or performance goals that they wish to apply and maintain from this training—increasing perception of determination.	The trainees feel motivated due to this perceived determination and develop the best ways to achieve and maintain such goals: Setting the skills maintenance goal, based upon the training, and identifying potential risks of slips.	By identifying potential threats to transfer, the trainees define the advantages and disadvantages of using the skills at work in order to keep motivated.
Operationalization	Trainees setting specific training goals and activities related to how they deal with the challenges in achievement such goals, when addressing difficulties in transfer. We expect to find evidence of this in the survey (trace evidence) which we asked trainees to complete after having attended the training.	Goal-oriented reasons that stimulated training application. There might be evidence in the form of trace evidence or account evidence.	Aspects that the trainees absorb during the training itself, that enable them to get focused in their efforts to apply and maintain from the training. This evidence could take the form of account evidence.	Trainees feeling motivated to reach a goal and actions are carried out to implement the training. We expect that this evidence will mainly be account evidence.	Trainees contrasting approaches about using/not using the skills at work, identifying potential threats to the training application and some ways to resolve it, and formulating advantages of its applicability. We expect to mostly find account evidence here.

	Coping with slips	Networking	Slip prediction	
	Part 5a	Part 5b	Part 6	
Theorization	Trainees discuss and learn certain methods or tools to avoid or overcome some kind of obstacles in applying the training content at work [anticipation].	Trainees understand the difference between training and job context, so they create a support network for transferability.	Trainees predict some kind of slips in transfer by monitoring past experiences of slip and relapse [anticipation] and the present environmental situations.	
Operationalization	Trainees dealing with challenging situations in the application of training to the job, as well as communication exchange about ways to overcoming it. We mainly expect to find account evidence for this.	Active supportive networks created by trainee in order to get transferability. We expect to find account evidence that could confirm this proposition.	Trainees’ reflections from past slips experiences and relapse as a way to anticipate such situations. This evidence can take the form of account evidence of the employees identifying some obstacles that arise when attempting to apply the training.	

	Coping strategies	Monitoring and self-rewards	OUTCOME	
	Part 7	Part 8		
Theorization	Based on coping methods, the trainees applies a threat coping strategy to this “predicted slip” by selecting only appropriate steps to increase skills retention and generalization [e.g., applying skills in the appropriate setting, reducing interfering and unproductive emotions; retain self-confidence, and diagnose support skills needed to maintain training].	The trainees monitor the process of skills transfer (self-monitoring if performance; self-evaluation against goal; self-reaction with self-efficacy) and creates meaningful self-rewards for skill retention.	Effective employee training transfer is the application or use of the learned knowledge (content, skills or attitudes) acquired in a training program to the job by trainees, which is maintained over a period of time. Learned content and skills are applied on the job context and maintained over time (routine).	
Operationalization	Trainees dealing with slipping up and overcoming threats to generalization, by reducing interfering and unproductive emotions and applying skills in the appropriate setting. We mostly assume that the evidence here will be account evidence. If the trainees have formalized this strategy, there could be a small chance we will find some trace evidence.	Activities performed by the trainees related to self-monitoring of their own performance and some sort of self-rewards for skill retention. We expect that this will mostly be account evidence in the form of the trainees discussing what helps them in implementing the training. We can also find trace evidence in the survey on how the trainee experiences the training.	The employees apply the learned content and skills and they are maintained on the job. Evidence for this can take the form of pattern evidence (the survey) or account evidence.	

Source: Alamos-Concha et al., 2021.

By unpacking this process from a system-level approach, we obtained an in-depth and nuanced understanding about the actual operation of the “self-management intervention” mechanism in a typical case. As such, this provided the appropriate evidence to validate the existence of a causal process between the observed configuration and the outcome.

Beyond the selection of one single typical case, one could equally select and compare two typical cases, which can logically increase confidence in (parts of) a particular mechanism. If one would want to boost confidence in the above-sketched “self-management intervention” mechanism, an analysis of case N2 would be an evident choice. The case shares the same configuration as case B3 in term 4. Given these similarities, N2 could thus provide cumulative evidence about the existence of the theorized mechanistic relationship and help us fine-tuning the mechanistic process. Especially if theory-building or theory-testing is at stake, this would be a logical scenario to consider.

Proceeding from internal validation to generalization at the within-case level, the question is whether context-sensitive mechanistic claims hold external validity toward other cases? Putting it differently, and applying it to our case: can we generalize the observed “self-management intervention” mechanism to other cases beyond B3, N2, and beyond term 4?

Acknowledging the contextually bounded reality of mechanisms, several strategies can be considered, again depending on one’s analytical goals. One possible strategy that has not received much attention in the literature entails the isolation of key condition(s), which we also coin as the “cross-term strategy.” It is here that the combination of QCA and PT can be exploited to the fullest. Elaborating further on the “self-management intervention” observed mechanism, we previously identified “relapse prevention-goal setting” as a condition triggering its functioning. Table 4 presents the conservative solution in a different fashion, and helps us clearly identifying the five cases sharing this condition: beyond the already examined cases B3 and N2, the mechanism can also be potentially present in J3, V2, and T1. These five cases all serve as so-called typical cases. Rather than investigating these three other cases in-depth, which could be beyond the scope of a research project, a better understanding of the contexts in which this mechanism operates can also help generalizing across cases, at least when assuming that cases sharing the same contexts and isolated conditions display mechanistic homogeneity. Recall that the four contextual parameters included in the QCA are “identical elements,” “training programme as active learning method,” “autonomy” and “balanced workload.” As it stands, V2, J3, N2, and B3 share the same context of “training programme as active learning method,” while cases N2, B3, V2, J3, and T1 share the context “autonomy.” From a cross-case lens, both contexts are equifinal for the cases V2, J3, N2, and B3. For T1 only “autonomy” is present as a contextual parameter. If we want to generalize “self-management intervention” as a mechanism, more insight in the functioning of the surrounding contextual conditions is essential. More specifically, one needs to sort out how these contexts are operating and whether they are really enablers for the well-functioning of the mechanism at stake. If that turns out to be the case, one can cautiously argue that self-management intervention also serves as a possible mechanism in explaining training transfer effectiveness in V2, J3, and/or T1.

**Table 4.** Overview of Grouped Cases Matching Common Conditions and Contexts.

Cause/context	Training program as active learning method	Identical elements	Autonomy	Balanced workload
Peer support and supervisor support	B2; K2		-	¬
Supervisor support	B2; K2; N2; B3			
Peer support	B1; K2; M1; D1; S2	B1; K2; M1; D1	T2	
Supervisor and relapse prevention setting goals	N2; B3			
Relapse prevention setting goals	V2; J3; N2; B3		N2; B3; V2; J3; T1	-
Sense of urgency and relapse prevention setting goals	T1			¬
Sense of urgency	W1	¬	¬	W1

Note: In the given context, the symbol “-” means “it does not matter,” while the symbol “¬” indicates “absent.”

Validity is logically presented as a first criterion, as it will always precede generalization. Self-evidently, if one wants to generalize, one needs to have confidence that a mechanism is indeed operative in specific contexts. With validity as analytical priority, typical cases with membership scores in the focal conjunct (FC) and outcome Y that are as similar as possible should first be selected.

When generalization of mechanistic findings is at stake, multiple typical cases (preferably more than two) covered by the same term can be scrutinized. Suitable cases should share the same causal conditions (to reduce the risk of causal heterogeneity at the cross-case level) and the same contexts (to reduce the risk of mechanistic heterogeneity at the level of the process). If the mechanism proves to be operative in these cases, it can be safely assumed that other cases covered by the same term, and sharing the same contexts, also display such a mechanism without the need to collect additional empirical evidence.

## Discussion and Conclusion: The Value of the QCA-PT Combination in View of Internal and External Validity

Combining QCA and PT within a multimethod design proves to be a powerful tool for knowledge creation in terms of identifying both *what* worked (core conditions) and *why* it worked (causal mechanisms) in a given policy or intervention. Within this design, QCA enables systematic cross-case level, whereas PT provides a close-up look at how a given process took place in a reduced number of cases. Thus, as illustrated by our real-life example, this combination of methods proves useful for increasing both the internal and external validity.

More specifically, with QCA we identify, in a systematic and transparent manner, different pathways that lead to a given outcome (equifinality). Each pathway contains a given number of cases, and is at least partly exclusive as some cases can be members of different pathways. In the real-life example we analyzed, the QCA solution provides an explanation for the positive cases of “training transfer effectiveness” (the outcome of interest). This explanation may lead to “modest” or “bounded’” generalization and may also contribute to middle-range theorizing (Ragin, 1987, 2014; Rihoux & Ragin, 2009). However, when we move to the within-case level, aiming for (empirical) generalization gets particularly tricky, especially when the QCA solution includes many terms (i.e., key combinations of conditions) comprising only few cases. The risk is then to come up with a long list of case-specific “explanations,” which may then simply come close to multiple parallel case descriptions, losing the whole added value of cross-case generalization.

In such a scenario, researchers may wish to resort to the “cross-term” strategy introduced in this paper. In essence, it consists in isolating a condition that is expected to be important to trigger a process. This expectation should ideally be both theory-informed and—perhaps more importantly—grounded in empirical observation. In concrete terms: the researcher gradually uncovers such a potential trigger condition during the QCA protocol which should involve regular iterations with case-based knowledge, as part of a planned multimethod design (Rihoux & Ragin, 2009; Rihoux et al., 2021) as discussed in the introduction. This approach as advocated in our contribution involves an alternative way of analyzing/reading a QCA solution, which offers much potential for multimethod research purposes—also beyond the field of evaluation. As explained above, the strategy basically involves scanning in which terms a particular condition of interest is present. On this basis, cases can subsequently be grouped per common contexts. One may then, via PT, unpack the functioning of such a condition for the mechanistic process in those cases that hold most theoretical relevance for the research. If the condition turns out to be operative (the concrete proof being provided via the PT analysis), one may assume to find the same mechanistic process in the group of cases sharing that same condition and same contexts.

Such a strategy could lead to a quite fundamental criticism: is it actually *relevant* to isolate one specific condition for the purpose of within-case analysis (via PT)? Indeed this may run against the QCA foundational “configurational” assumption according to which conditions operate in combination. This is a valid remark, but we do believe that the generalization technique we propose brings some added value particularly when the QCA solution terms comprise only a few cases per term, and when this QCA solution also displays multiple pathways comprising several conditions and contexts. The technique then in essence boils down to collecting evidence on the functioning of *micro-mechanisms* (e.g., in the real-life evaluation presented in Table 2: “self-management intervention” is a case in point) triggered by single causal conditions (“relapse prevention-goal setting”)—and then putting micro-mechanisms, trigger conditions and contexts together to uncover a complex macro-mechanism (as a system)—as displayed in Figure 3. Micro-mechanisms may in fact act in parallel or in sequence depending on the nature of the conditions and contexts at play (see Figure 3). Once robust evidence on the existence of micro-mechanisms is collected, one can, in a further stage, analyze how they are connected, and in which sequence they transmit causal forces in producing the outcome.

The QCA-PT multimethod design we are proposing clearly demonstrates the added value of exploiting QCA when engaging in system-level PT. As explained above, a system-level approach to PT by nature implies quite an investment for researchers, not least in terms of time and resources, because of the depth of empirical evidence one needs to collect. Indeed, PT best practice requires a very careful strategy to empirically trace each of the constituent parts of a mechanism, that is, by meticulously observing the empirical fingerprints left by the activities of entities in each part of the process (Beach & Pedersen, 2016, 2019).

An example of a fingerprint is the evidence for the B3 case studied in the PT part. For instance, to confirm the theory that part 1 of the causal mechanism “self-management intervention” is operative, we need to observe some of the fingerprints operationalized in Table 2. We expected to find evidence in the empirical record of goal-oriented reasons that stimulated training application. When B3 was asked in an open question what had stimulated them to apply the training contents, they replied, “*my conviction that my communication can be better so that my message gets across clearer and more respectfully to my staff members/colleagues*.” (Alamos-Concha et al., 2021).

Given these requirements, the number of potentially interesting mechanisms to trace will often exceed the possibilities of a single researcher/project. This is precisely where QCA comes into play: when exploited as a preceding method (before system-oriented PT), it provides the systematic leverage to identify, in a theory- and case-informed way, key combinations of conditions leading to the outcome of interest, and to “handpick” specific cases, as such supporting the external validity and generalization of the PT findings. Our contribution can thus be seen as a complement to available multimethod research guidance, both in the field of evaluation and in other fields where the QCA-PT sequence may bring a similar added value. There is certainly room for further refinement of the design we have developed. One particular avenue would be to examine more closely the “negative” cases, that is, those in which a given causal mechanism quite likely failed to operate (“break” in the hypothesized causal chain). We nonetheless believe we have built a robust protocol that enables one to address both “why” and “how” questions: WHY is a policy intervention successful in some cases and not in others? What are pathways to success? And HOW does this actually unfold in individual cases? What are the core aspects in the process that make the intervention work? In the above-discussed design, QCA boosts external validity by enabling empirical generalization, while PT boosts internal validity by providing “deep” case-grounded evidence.

## Note

1 “FC is the conjunct for which we want to find out whether it makes a difference for the mechanism M” (Schneider & Rohlfing, 2019, p. 258).

## References

[bibr1-0193841X251352020] Álamos-Concha P. Cambré B. Foubert J. Pattyn V. Rihoux B. Schalembier B. (2021). Impact evaluation ESF intervention training in companies. Department of Work and Social Economy. https://www.vlaanderen.be/publicaties/impactevaluatie-esf-interventie-opleidingen-in-bedrijven-what-drives-training-transfer-effectiveness-and-how-does-this-transfer-work (Accessed 22 June 2024).

[bibr2-0193841X251352020] Alamos-Concha P. Pattyn V. Rihoux B. Schalembier B. Beach D. Cambré B. (2022). Conservative solutions for progress: On solution types when combining QCA with in-depth process-tracing. Quality and Quantity, 56(4), 1965–1997. 10.1007/s11135-021-01191-x

[bibr3-0193841X251352020] Beach D. Camacho G. Siewert M. (2022). Going beyond the single case: Comparative Process Tracing as a tool to enable generalizations about causal processes. : Annual Meeting of the American Political Science Association.

[bibr4-0193841X251352020] Beach D. Pedersen R. B. (2016). Causal case study methods: Foundations and guidelines for comparing, matching, and tracing. University of Michigan Press.

[bibr5-0193841X251352020] Beach D. Pedersen R. B. (2019). Process-tracing methods: Foundations and guidelines. : University of Michigan Press.

[bibr6-0193841X251352020] Befani B. (2016). Pathways to change: Evaluating development interventions with qualitative comparative analysis (QCA). Rapport till Expertgruppen För Biståndsanalys (EBA). Disponible en. https://eba.se/wp-content/uploads/2016/07/QCA_BarbaraBefani-201605.pdf

[bibr7-0193841X251352020] Befani B. Sager F. (2006). QCA as a tool for realistic evaluations. In Rihoux B. Grimm H. (Eds.), Innovative comparative methods for policy analysis (pp. 263–284). Springer.

[bibr8-0193841X251352020] Befani B. Stedman-Bryce G. (2017). Process tracing and Bayesian updating for impact evaluation. Evaluation, 23(1), 42–60. 10.1177/1356389016654584

[bibr9-0193841X251352020] Berg-Schlosser D. De Meur G. Rihoux B. Ragin C. (2009). Qualitative comparative analysis (QCA) as an approach. In Rihoux B. Ragin C. C. (Eds.), Configurational comparative methods. Qualitative Comparative Analysis (QCA) and related techniques (pp. 1–18). : Sage.

[bibr10-0193841X251352020] Blackman T. Wistow J. Byrne D. (2013). Using qualitative comparative analysis to understand complex policy problems. Evaluation, 19(2), 126–140. 10.1177/1356389013484203

[bibr11-0193841X251352020] Goertz G. (2006). Social science concepts: A user’s guide. Princeton University Press. 10.2307/j.ctvcm4gmg

[bibr12-0193841X251352020] HM Treasury . (2020). Magenta Book supplementary guide: Handling complexity in policy evaluation. Disponible en. https://www.gov.uk/government/publications/the-magenta-book

[bibr13-0193841X251352020] Johnson R. B. Onwuegbuzie A. J. Turner L. A. (2007). Toward a definition of mixed methods research. Journal of Mixed Methods Research, 1(2), 112–133. 10.1177/1558689806298224

[bibr14-0193841X251352020] Krupnik S. Szczucka A. Woźniak M. Pattyn V. (2023). The potential of consecutive qualitative comparative analysis as a systematic strategy for configurational theorizing. Evaluation, 29(4), 451–467. 10.1177/13563890231200292

[bibr15-0193841X251352020] Lemire S. Peck L. R. Porowski A. (2020). The growth of the evaluation tree in the policy analysis forest: Recent developments in evaluation. Policy Studies Journal, 48(S1), S47–S70. 10.1111/psj.12387

[bibr16-0193841X251352020] Machamer P. (2004). Activities and causation: The metaphysics and epistemology of mechanisms. International Studies in the Philosophy of Science, 18(1), 27–39. 10.1080/02698590412331289242

[bibr17-0193841X251352020] Machamer P. Darden L. Craver C. F. (2000). Thinking about mechanisms. Philosophy in Science, 67(1), 1–25. 10.1086/392759

[bibr18-0193841X251352020] Pattyn V. (2023). Qualitative comparative analysis. In LIEPP methods brief 39: Sciences.

[bibr19-0193841X251352020] Pattyn V. Álamos-Concha P. Cambré B. Rihoux B. Schalembier B. (2022). Policy effectiveness through configurational and mechanistic lenses: Lessons for concept development. Journal of Comparative Policy Analysis: Research and Practice, 24(1), 33–50. 10.1080/13876988.2020.1773263

[bibr20-0193841X251352020] Pattyn V. Molenveld A. Befani B. (2019). Qualitative comparative analysis as an evaluation tool: Lessons from an application in development cooperation. American Journal of Evaluation, 40(1), 55–74. 10.1177/1098214017710502

[bibr21-0193841X251352020] Pawson R. (2008). Causality for beginners. ESRC/NCRM research methods festival. Disponible en. https://eprints.ncrm.ac.uk/245/

[bibr23-0193841X251352020] Ragin C. C. (1987). The comparative method: Moving beyond qualitative and quantitative strategies. University of California Press.

[bibr24-0193841X251352020] Ragin C. C. (2014). The comparative method: Moving beyond qualitative and quantitative strategies. University of California Press.

[bibr25-0193841X251352020] Raimondo E. (2020). Getting practical with causal mechanisms: The application of process-tracing under real-world evaluation constraints. New Directions for Evaluation, 2020(167), 45–58. 10.1002/ev.20430

[bibr26-0193841X251352020] Raimondo E. (2023). Process tracing. In LIEPP methods brief 41. Sciences Po.

[bibr27-0193841X251352020] Rihoux B. Álamos-Concha P. Lobe B. (2021). Qualitative comparative analysis (QCA): An integrative approach suited for diverse mixed methods and multimethod research strategies. In Onwuegbuzie A. J. Johnson R. B. (Eds.), The Routledge reviewer’s guide to mixed methods analysis (pp. 185–198). Routledge.

[bibr28-0193841X251352020] Rihoux B. Ragin C. C. (Eds.), (2009). Configurational comparative methods. Qualitative comparative analysis (QCA) and related techniques. Sage.

[bibr29-0193841X251352020] Schneider C. Q. Rohlfing I. (2019). Set-theoretic multimethod research: The role of test corridors and conjunctions for case selection. Swiss Political Science Review, 25(3), 253–275. 10.1111/spsr.12382

[bibr36-0193841X251352020] Schneider C. Q. Wagemann C. (2012). Set-Theoretic Methods for the Social Sciences: A Guide to Qualitative Comparative Analysis. Cambridge: Cambridge University Press.

[bibr30-0193841X251352020] Stame N. (2004). Theory-based evaluation and types of complexity. Evaluation, 10(1), 58–76. 10.1177/1356389004043135

[bibr31-0193841X251352020] Stern E. Stame N. Mayne J. Forss K. Davies R. Befani B. (2012). Broadening the range of designs and methods for impact evaluations. Department for International Development (DFID).

[bibr32-0193841X251352020] Vaessen J. Lemire S. Befani B. (2020). Evaluation of international development interventions: An overview of approaches and methods: World Bank. Independent Evaluation Group.

[bibr33-0193841X251352020] Verweij S. Gerrits L. (2013). Understanding and researching complexity with qualitative comparative analysis: Evaluating transportation infrastructure projects. Evaluation, 19(1), 40–55. 10.1177/1356389012470682

[bibr34-0193841X251352020] Waldner D. (2014). What makes process tracing good? In Bennett A. Checkel J. (Eds.), Process tracing: From metaphor to analytic tool (Strategies for social inquiry). Cambridge University Press.

[bibr35-0193841X251352020] Wauters B. Beach D. (2018). Process tracing and congruence analysis to support theory-based impact evaluation. Evaluation, 24(3), 284–305. 10.1177/1356389018786081

